# Manganese superoxide dismutase plays an important role in the inflammatory process and predicts disease severity and activity in patients with ulcerative colitis

**DOI:** 10.1111/apm.12192

**Published:** 2014-02-01

**Authors:** Taro Ikumoto, Shinichi Hayashi, Shigeki Tomita, Shigeharu Miwa, Hiroyuki Mitomi, Takahiro Fujimori, Johji Imura

**Affiliations:** 1 Department of Surgical and Molecular Pathology, Dokkyo University School of MedicineMibu, Japan; 2 Department of Diagnostic Pathology, Graduate School of Medicine and Pharmaceutical Sciences, University of ToyamaToyama, Japan

**Keywords:** MnSOD, biomarker, ulcerative colitis, immunohistochemistry, patient outcome

## Abstract

The aim of this study was to investigate the expression pattern of manganese superoxide dismutase (MnSOD) in relation to inflammatory factors in ulcerative colitis (UC) and characterize this enzyme as a newly identified biomarker potentially linked to disease pathogenesis of UC. MnSOD expression was analyzed immunohistochemically in 48 formalin-fixed and paraffin-embedded specimens from patients with UC who had undergone endoscopical biopsy. MnSOD expression was observed in vascular endothelium, macrophages, and polymorphonuclear leukocytes within lamina propria of inflamed mucosa. The patients who did not express MnSOD tended to have stabilization of symptoms, but accompanied with status of inflammation. The MnSOD expression pattern was strongly correlated with disease type. MnSOD was expressed in polymorphonuclear leukocytes of all disease types, but cases of chronically counting and exacerbation type had particularly high frequency of immunopositive cells. MnSOD expression in macrophages was frequently observed in cases of symptom remaining type. The cases with MnSOD expression in the vascular endothelium showed a tendency to express in relapse-remission and exacerbation of symptoms. Immunohistochemical evaluation for MnSOD expression may be useful for predicting disease severity and activity in patients with UC.

Inflammatory bowel disease (IBD) is a complex inflammatory disease of the gastrointestinal tract with unknown cause that lacks reliable biomarkers to monitor disease progression and response to treatment. Ulcerative colitis (UC) and Crohn’s disease are the two major forms of IBD. UC is a form of IBD characterized by damage of the large bowel mucosa. The roles played in IBD pathogenesis by the molecular factors known to interact with components of the several mechanisms involved in the inflammatory reaction have not been completely described. The most widely accepted hypothesis of IBD pathogenesis is that the mucosal immune system mounts an aberrant response toward luminal antigens such as dietary factors and/or commensal bacteria in genetically susceptible individual 1–5. As IBD is characterized by recurrent flare-ups interspersed with clinical remission, reliable and practical indices for evaluating and monitoring the disease severity are required. The disease activity of IBD is usually assessed by a combination of clinical symptoms and laboratory data, such as leukocyte count, serum C-reactive protein level, and erythrocyte sedimentation rate 6–9. However, these indices are not specific for IBD and do not always correlate with the severity of the intestinal inflammation 7,9,10. By contrast, colonoscopy and mucosa biopsy are regarded as the most accurate and objective measures of colorectal inflammation, and are the ‘gold standard’ in IBD diagnosis 9,11. Matt’s classification of the pathological findings from the mucosal biopsy has recently been proposed as a method to evaluate degree of inflammatory activity and is now in practice. Although this classification is useful for evaluation of the degree of inflammatory activity at one time point, it is difficult to predict the clinical outcome for a patient. Accordingly, reliable biomarkers to monitor disease progression and response to treatment should be established.

From previous basic studies of the inflammatory mechanisms in various organs, the involvement of several kinds of factors was clarified. Among them, the group of nitric oxygens, represented by superoxide, has been considered until now as aggressive mediators in inflammatory lesions. The superoxide dismutases (SODs) catalyze the reaction 20_2_^−^ + 2H^+^ to H_2_O_2_ + O_2_, thus eliminating superoxide radicals. Hydrogen peroxide is further reduced to H_2_O + O_2_ by catalase and glutathione peroxidase. In eukaryotes, three SODs have been described. It was reported recently that manganese superoxide dismutase (MnSOD) is one of the converting enzymes of these active oxygen groups that participates in the inflammatory foci. MnSOD is a nuclear-encoded mitochondria matrix protein 12. Induction of MnSOD is protective against radical-mediated damage as well as against tumor necrosis factor alpha (TNF-α) and interleukin-1 (IL-1) cytotoxicity 13,14, implicating a free radical-mediated cytotoxicity mechanism. Thus, regulation of MnSOD may act as a protective defense against cytokine toxicity and mitochondrial oxygen radical production. In intestinal epithelial cell lines, MnSOD expression is regulated by the cytokines TNF-α, IL-lα, and IL-1β, as well as by bacterial endotoxin 15.

To the best of our knowledge, no studies thus far have reported the evaluation of MnSOD immunoexpression accompanied by clinicopathological findings in UC. Given the function of MnSOD in inflammatory reaction, it may be one of the important active players in UC pathogenesis. In the present study, we examined MnSOD expression by immunohistochemistry in mucosal biopsies obtained from UC patients. Furthermore, we studied whether evaluation of MnSOD expression together with clinicopathological findings is useful to predict disease severity and activity of UC.

## Materials and Methods

### Patients and tissue processing

In this study, mucosal biopsies previously obtained from 48 Japanese adult patients with UC without systemic comorbidities and stored in the archival records of our department were included. Furthermore, 15 non-UC peoples, as control group, who enforced the lower intestinal endoscopy for the purpose of general examination, were examined. At the time of collection, the patients’ demographics, symptoms, treatment, and endoscopic grading were recorded. Inflammation status was separated into two types, relapse-remission (RR type) and chronically continuing (CC type) types. The patient outcome was classified as exacerbation (E type) or symptom remaining type (SR type). Biopsies were obtained at first diagnosis. Histological disease activity in each section (stained with hematoxylin and eosin) was assessed using the Matt’s histological grading system from 1 to 5 16 by two independent observers (KI and JI).

All the procedures followed were in accordance with the ethical standards of the responsible committees (institutional and national) on human experimentation. Informed consent was obtained from all patients at the time of biopsy, and the study was approved by our local Ethics Committee.

All samples were archived formalin-fixed and paraffin-embedded tissue, which were cut into 5 μm sections and rehydrated for immunohistochemical analysis.

### Immunohistochemical study

The rehydrated sections were treated in 0.01 M citrate buffer (pH 6.0) and microwave heating (400 W, 95 °C; MI-77; Azumaya, Tokyo, Japan) for 40 min to facilitate antigen retrieval. The sections were then pretreated with 0.3% H_2_O_2_ in methanol at room temperature to quench endogenous peroxidase activity. This was followed by blocking with Protein Block Serum-Free (Dako, Carpinteria, CA, USA) for 30 min, and incubation with anti-MnSOD polyclonal antibody (dilution 1:400, Fukuyama Rinsho, Tokyo, Japan) for 1 h. Thereafter, the sections were incubated with biotinylated secondary antibody for 15 min, washed with PBS, and treated with peroxidase-conjugated streptavidin (Dako) for 20 min. Finally, the sections were visualized by incubating in 3,3′-diaminobenzidine tetrahydrochloride with 0.05% H_2_O_2_ (Liquid DAB+Substrate Chromogen System; Dako) for 3 min and then counterstained with Carazzi’s hematoxylin. Two independent observers examined the specimens in a blinded manner.

### Statistical analysis

Data were analyzed with StatView software (Japanese version, Hulinks, Tokyo, Japan). Analyses between two groups were compared with Student’s *t*-test. Differences were considered statistically significant when p < 0.05.

## Results

### Correlation between inflammatory status, patient outcome, and clinicopathological findings

The endoscopical and/or histological inflammatory findings were not recognized in this control group. Patient characteristics are summarized in Table 1. The number of male and female patients was similar, and their average age was 34.2 years (range, 16–62 years). The biopsy specimens were consecutively collected from the rectum to cecum. With regard to the distribution of the lesions, 17 cases were taken from the left colon (35%), and 31 samples were of the total colonic type (65%). The disease duration widely ranged from 1 to 34 years, and the mean was 9.2 years. Classification of the cases according to Matt’s grade was as follows: four specimens (8%) were designated as grade 1; seven (16%) were grade 2; 14 (29%) were grade 3; 13 (27%) were grade 4; and 10 (20%) were grade 5. Regarding the inflammatory status and patient outcome, 31 cases (65%) were of RR type and 17 (35%) of CC type, and 15 patients (31%) were E type and 33 (69%) were SR type, respectively. As for all UC patients, the treatment with medication was provided. The salicylazosulphapyridine as non- steroid treatment for UC was given to the patients. The combination therapy with the steroid (prednisolone) was only some patients (6/48:12%). The significant differences were not recognized in the kind of medication, regimen, schedule, and dosage (Table 1). There were no statistical significances between the status of inflammation, or the type of patients outcome and the distribution of gender, age, region, distribution of lesion, disease duration, and Matt’s grade (Table 2).

**Table 1:** Summary of clinical and pathological information on the 34 patients with ulcerative colitis included in our study

Characteristic	
No. of patients	48
Type of distribution	
Left colonic	17
Total colonic	31
Age of onset	
Median age (range)	34.2 (16–62)
Disease duration	
Median year (range)	9.2 (1–34)
Matt’s grade	
Grade 1	4
Grade 2	7
Grade 3	14
Grade 4	13
Grade 5	10
Medication	
Steroid alone	42
Combination	6
Status of inflammation	
Relapse-remission (RR)	31
Chronically continuing (CC)	17
Type of patient outcome	
Exacerbation (E)	25
Symptom remaining (SR)	23

**Table 2:** Correlation between two typing clinical course and clinicopathological factors

Clinicopathological factor	Status of inflammation			Type of patient outcome		
	RR	CC	p	E	SR	p
Gender						
Male	17	10	n.s.	14	13	n.s.
Female	14	7		11	10	
Age of onset						
Median (range)	34 (22–62)	26 (16–52)	n.s.	35 (18–57)	30 (16–62)	n.s.
Type of distribution						
Left colonic	7	10	n.s.	8	9	n.s.
Total colonic	24	7		17	14	
Disease duration						
Median year (range)	8.7 (6–18)	8.08 (1–34)	n.s.	8.5 (1–24)	7.8 (2–34)	n.s.
Matt’s grade						
Grade 1	2	2	n.s.	3	1	n.s.
Grade 2	4	3		5	2	
Grade 3	10	4		6	8	
Grade 4	8	5		6	7	
Grade 5	6	4		5	5	

RR, relapse-remission; CC, chronically continuing; E, exacerbation; SR, symptom remaining; n. s.: not significant.

### MnSOD immunohistochemical study

**Figure 1:**
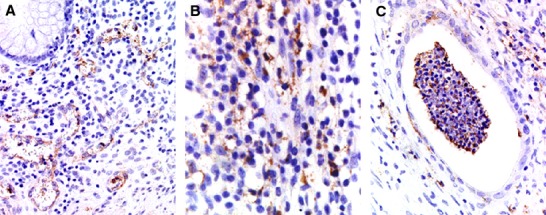
Comparison of immunohistochemical manganese superoxide dismutase (MnSOD) expression profiles in the inflammatory mucosa obtained from patients with UC: a pronounced positive reaction for MnSOD in vascular endothelium (A), macrophages (B), and polymorpholeukocytes (C).

**Figure 2:**
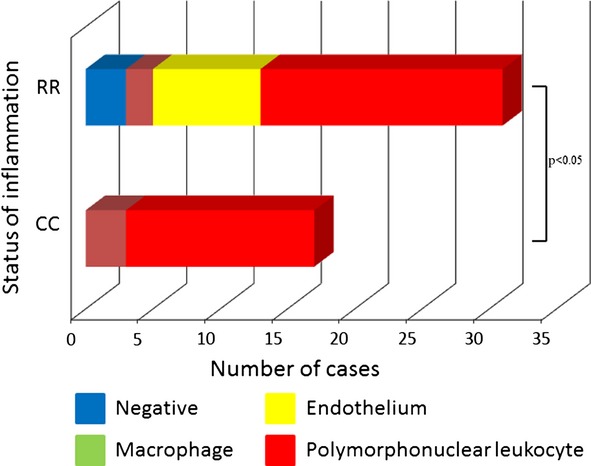
Correlation between status of inflammation and expression pattern of manganese superoxide dismutase.

**Figure 3:**
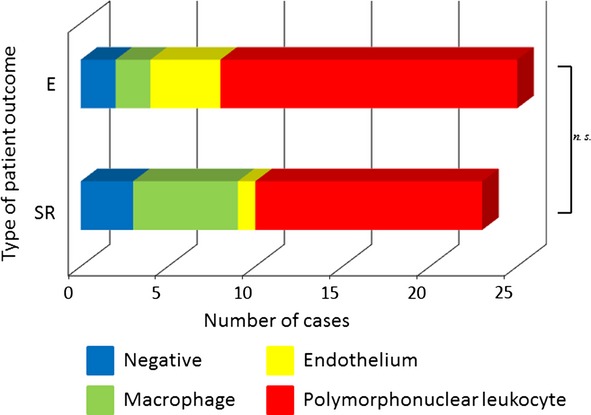
Correlation between type of patient outcome and expression pattern of manganese superoxide dismutase.

MnSOD immunoreactivity was positive in 45 (93%) cases of UC patient group. On the other hand, positive reaction was observed only in two cases (13%) of the control group. This enzyme was expressed in the vascular endothelium, macrophages, and polymorphonuclear leukocytes within lamina propria of inflamed mucosa, but not in epithelial cells. We also observed cytoplasmic localization of this protein in all of these cells. Comparison of the immunohistochemical pattern is shown in Fig. 1. No significant difference between the expression pattern of MnSOD and Matt’s grade was observed. However, MnSOD expression can be divided into four patterns according to inflammatory status and patient outcome. Namely, although MnSOD immunopositivity in polymorphonuclear leukocytes was observed in most cases, this expression pattern was more frequently found in cases of CC and E type. Cases of SR type were shown to contain a greater number of immunopositive macrophages. The cases with high MnSOD expression in the vascular endothelium tended to be of RR and E type. The patients who did not express MnSOD tended to be of RR or SR type (Figs 2 and 3).

## Discussion

Ulcerative colitis affects the large bowel, particularly rectum and colon, in which it presents characteristic ulcers 17. In the intestinal mucosa, the lamina propria is infiltrated by abundant inflammatory cells, while the epithelium undergoes cycles of destruction and repair by regeneration of basal cells 18. The inflammatory processes include different phases, which are associated with many factors and mechanisms. In the present study, we revealed the expression of MnSOD in the inflamed mucosa from patients with UC. The expansion of research involving nitric oxide (NO) has shown that this small molecule has diverse roles in normal physiology and pathological conditions 19–23. The function of NO in colonic inflammation, however, remains unclear. The detrimental or beneficial effects of NO that have been observed in the various animal models of colitis may reflect several factors in addition to quantity of NO. Further investigations into the roles of NO in colitis will likely require specific inhibitors of constitutive NOS and iNOS as well as an evaluation of the redox state and the levels of MnSOD. Excessive NO production by an isoform of NO synthase that can be induced by inflammatory stimuli leads to changes in vascular permeability and tissue injury. Boughton-Smith et al. examined the activity of NO synthase in mucosa and muscle from patients with UC and Crohn’s disease 24. NO synthase activity in colonic mucosa of UC patients was about eightfold higher than in control group. In colonic muscle, there was no difference in NO synthase activity between UC patients and controls. In patients with Crohn’s disease, mucosal NO synthase activity did not differ from control values and activity in the colonic muscle was low. From these results, they proposed that active UC is associated with a substantial increase in Ca^2+^-independent NO synthase activity in the colonic mucosa, characteristic of the inducible form of the enzyme. It may be that one of these enzymes is MnSOD. They inferred that the activity of NO synthase is induced by infiltrating inflammatory cells in colonic mucosa of UC, resident macrophages, vascular endothelial, vascular smooth muscle, mucosal epithelial, or other mucosal cells. These inflammatory cells may be activated by luminal bacterial products after epithelial cell disruption or by the intramucosal release of cytokines such as TNF-α or IL-1 25. NO or a cytotoxic NO metabolite generated by this induced NO synthase activity is likely to contribute to the vasodilation, increased vascular permeability, and tissue injury seen in active UC. Furthermore, active UC is associated with increased cytokine production, mucosal vasodilation, and enhanced vascular permeability 26. Interestingly, there is a lack of increase in mucosal NO synthase activity in patients with Crohn’s disease 25. Thus, different underlying mechanisms are likely involved in UC and Crohn’s disease.

Biological markers have been studied in IBD to monitor disease activity and predict the risk of relapse 27. An assessment of disease activity in patients with IBD can be carried out using clinical disease activity indices, endoscopic indices, serum markers, and fecal markers 28. Clinical indices give an indirect measurement of disease activity, and may not accurately predict inflammatory activity found by endoscopic and histological examinations. The development of a new marker specific for mucosal inflammation would be helpful for enhancing the accuracy of monitoring disease severity and activity. In the present study, we showed MnSOD expression in vascular endothelium, macrophages, and polymorphonuclear leukocytes in mucosal biopsy specimens obtained from UC patients. In the control group, expression of MnSOD was observed in only two cases (13%). The patients who did not express MnSOD tended to have stabilization of symptoms, but accompanied with status of inflammation. MnSOD expression in polymorphonuclear leukocytes was observed in all disease types, but the cases with CC and E type showed a particularly high frequency of this expression pattern. On the other hand, cases with frequent expression of this enzyme in macrophages were prone to alternating SR type. In particular, high MnSOD expression in the vascular endothelium had a tendency to occur in cases with RR and E type. From these results, MnSOD expression can be divided into four patterns according to inflammatory status and patient outcome.
